# Development, Characterization and Resveratrol Delivery of Hollow Gliadin Nanoparticles: Advantages over Solid Gliadin Nanoparticles

**DOI:** 10.3390/foods12132436

**Published:** 2023-06-21

**Authors:** Duoduo Li, Zihao Wei, Xiaolong Li

**Affiliations:** College of Food Science and Engineering, Ocean University of China, Qingdao 266404, China

**Keywords:** gliadin nanoparticle, sacrificial template, hollow structure, resveratrol, delivery system, sustained release

## Abstract

Hollow nanoparticles have attracted extensive attention due to their advantages such as high loading capacity and superior stability. However, the complexity of the preparation process and harmfulness of the used raw materials have limited their application in the food field. Based on this, hollow gliadin nanoparticles (HGNPs) were developed using a Na_2_CO_3_ sacrificial template method. The findings of this study suggested that HGNPs could be regarded as a delivery system for resveratrol (Res) and they exhibited excellent delivery performance. Compared with solid gliadin nanoparticles (SGNPs), the HGNPs displayed smaller particle sizes, better physical stability, higher encapsulation efficiency, stronger resistance to ultraviolet light and a more sustained release of Res in the gastrointestinal tract. This work is of practical significance for the development and utilization of protein-based nanoparticles with hollow structures as a delivery system for sensitive bioactives.

## 1. Introduction

As a plant protein extracted from the by-products of wheat, gliadin is rich in essential amino acids for the human body and exhibits many favorable characteristics, such as amphiphilicity, foaming capacity, surface hydrophobicity and safety [1]. The amphiphilic structure of gliadin is composed of a hydrophilic central domain rich in glutamine as well as proline and two hydrophobic terminal domains containing many hydrophobic amino acids (phenylalanine, leucine, tryptophan, etc.), which is the premise of gliadin’s self-assembly into nanoparticles [2,3]. So far, solid gliadin nanoparticles have been extensively regarded as delivery carriers of bioactives substances [4,5]. It was reported that the solid gliadin nanoparticles prepared by an antisolvent precipitation method possessed excellent stability and encapsulation capacity [6]. In addition, gliadin-based nanoparticles can attach directly to the intestinal mucosa or bind to mucosal proteins through disulfide bonds, hydrogen bonds or hydrophobic interactions, thus, facilitating the absorption of loaded bioactive substances [7,8]. For example, Voci et al. (2022) reported that gliadin nanoparticles could prolong the residence as well as the release time of the loaded ascorbic acid in the digestive system, thereby promoting the absorption of ascorbic acid [9]. However, solid gliadin nanoparticles also manifested many drawbacks, including low encapsulation capacity and weak protective ability against the loaded bioactive substances.

In recent years, hollow nanoparticles (HNPs), a novel type of nanoparticles with cavities inside the shell, have received extensive attention from researchers [10,11]. The unique hollow structure of HNPs endows them with distinctive advantages, including a large specific surface area, favorable stability, low density and a high loading capacity, which also make them widely used in biomedicine, catalysis, energy and environment fields [12,13,14]. It was demonstrated that HNPs could be used as a potential carrier for certain drugs and enzymes [15]. For instance, Gupta et al. (2023) successfully developed hollow gold nanoparticles using calcium phosphate nanoparticles as templates to encapsulate horseradish peroxidase, which improved the activity and efficiency of horseradish peroxidase in cancer treatment [16]. In terms of serving as delivery carriers for bioactive substances, HNPs possess the following advantages over conventional solid nanoparticles [10,17,18]: (i) the internal cavity of HNPs can encapsulate more bioactive substances, which leads to higher encapsulation efficiency; (ii) the content of the biopolymers used is significantly reduced, which leads to lower bulk density; (iii) bioactive substances are encapsulated within the cavity of HNPs, which can provide better protection for them. However, the preparation process of HNPs is complicated and usually requires some extreme conditions (high temperatures, strong alkaline, strong acid, etc.). In addition, the use of non-food grade raw materials and organic solvents in the preparation process is harmful to the human body, thus limiting the application of HNPs in the food field.

Against this background, the hollow gliadin nanoparticles were developed using a simple and non-toxic sodium carbonate (Na_2_CO_3_) sacrificial template method. The Na_2_CO_3_ template could be easily removed with water and did not require harsh or extreme conditions. Furthermore, resveratrol was selected as the bioactive substance model to evaluate the performance and efficiency of hollow gliadin nanoparticles. The optimal conditions for the preparation of resveratrol-loaded hollow gliadin nanoparticles were initially investigated. The microstructure of nanoparticles was characterized by TEM. The FTIR and fluorescence spectroscopy were further performed to determine the interaction between resveratrol and gliadin. Furthermore, two types of gliadin nanoparticles (hollow and solid) were compared in terms of their structure, properties and release mechanisms. This research can facilitate the reasonable design and development of hollow gliadin nanoparticles as the delivery carriers of nutraceuticals.

## 2. Materials and Methods

### 2.1. Materials

Wheat flour was purchased from Qilu Biotechnology Co., Ltd. (Shandong, China). Resveratrol (trans, purity ≥99%) was obtained from Aladdin Biochemical Technology Co., Ltd. (Shanghai, China). Pepsin (3000 U/mg), pancreatin (4000 U/g), Tris and pig bile salt (the content of cholic acid ≥65%) were obtained from Solarbio Co., Ltd. (Beijing, China). All other chemicals were purchased from Sinopharm Chemical Reagent Co., Ltd. (Shanghai, China) and were analytical grade.

### 2.2. Extraction of Gliadin

The extraction of gliadin (Gli) referred to the method of Peng et al. (2017) [19]. In short, wheat flour was defatted by mixing with dichloromethane at a ratio of 1:10 (*w*/*v*) and stirring continuously for 2 h. After standing, dichloromethane (upper layer) was poured off. The above procedure was repeated 3 times. The lower solution was placed in a ventilation cabinet for airing so that the remaining dichloromethane could evaporate overnight at an ambient temperature to obtain defatted wheat flour. The degreased wheat flour was mixed with 70% (*v*/*v*) ethanol solution at 1:10 (*w*/*v*) under continuous agitation for 2 h to extract Gli. The resulting solution was then centrifuged at 10,000× *g* for 10 min. The obtained supernatant was refrigerated at 4 °C overnight and centrifuged again under the same conditions to further remove possible precipitates. Subsequently, the ethanol was removed using a rotary evaporator (N-1300, Eyela Instrument Company) at 65 rpm at 45 °C. The above solution was placed in deionized water and dialyzed in a dialysis bag (10,000 Da) at 4 °C for 3 days. Finally, the resulting solution was lyophilized for 2 d to gain Gli powder.

### 2.3. Preparation of Gliadin-Based Nanoparticles

#### 2.3.1. Preparation of Hollow Gliadin Nanoparticles (HGNPs) and Resveratrol-Loaded Hollow Gliadin Nanoparticles (Res-Loaded HGNPs)

Gli powder (500 mg) was dissolved in 10 mL of 70% (*v*/*v*) of ethanol solution, stirred for 30 min and then centrifuged at 10,000 rpm at 4 °C for 20 min. Subsequently, the supernatant was filtered with a 0.45 µm syringe to remove insoluble particles and obtain the Gli stock solution. The 2% Na_2_CO_3_ aqueous ethanol solution was prepared as the sacrificial template with reference to the approach of Li et al. (2019) [20]. After that, the above template solution was dropped into the Gli stock solution and stirred continuously for 30 min so that the Gli could be coated onto the surface of Na_2_CO_3_ particles. The above solution was injected into distilled water at 1:50 (*v*/*v*) and stirred continuously for 10 min. When water diffused into the core, Na_2_CO_3_ seeped out of the core due to its excellent solubility in water, thus, spontaneously forming HGNPs. In order to load resveratrol (Res), Res powder (250 mg, 100 mg, 50 mg or 25 mg) was added into the Gli stock solution (10 mL) and agitated until fully dissolved so that the Res/Gli mass ratio was 1:2, 1:5, 1:10 and 1:20. The subsequent preparation steps were the same as those for the preparation of HGNPs.

#### 2.3.2. Preparation of Solid Gliadin Nanoparticles (SGNPs) and Resveratrol-Loaded Solid Gliadin Nanoparticles (Res-Loaded SGNPs)

The Gli stock solution prepared in Section 2.3.1 was dropped into distilled water at a volume ratio of 1:50 (Gli stock solution: distilled water) and stirred continuously for 10 min to obtain SGNPs. To prepare Res-loaded SGNPs, 250 mg, 100 mg, 50 mg or 25 mg of Res powder were added into a 10 mL Gli stock solution, respectively. The mixed solution was then dropped into distilled water at 1:50 (*v*/*v*) with continuous agitation for 10 min.

### 2.4. Determination of Particle Size and ζ-Potential

The particle size, ζ-potential and polydispersity index (PdI) of gliadin nanoparticles were carried out by the Zetasizer (Malvern, Nano-ZS90, UK) based on dynamic light scattering (DLS) technology. All samples were equilibrated at 25 °C for 100 s inside the instrument before measurement. The data for each sample were obtained in triplicate.

### 2.5. Fourier Transform Infrared (FTIR) Spectroscopy

The lyophilized sample powder was mingled with dried spectral KBr powder (1:100, *w*/*w*) and pressed into thin sheets. The sheets were scanned using FTIR instrument (Thermo Scientific, Waltham, MA, USA). The spectral data were collected in the wavenumber range of 4000 cm^−1^ to 400 cm^−1^ with a resolution of 2 cm^−1^ for 64 times.

### 2.6. Fluorescence Spectroscopy

The fluorescence emission spectra of samples at a Res concentration of 20 μg/mL were determined by a fluorescence spectrophotometer (F-4600, Hitachi, Japan). The excitation wavelength was set to 306 nm. The spectra were collected from 320 nm to 510 nm. The spectral resolution of both the excitation and emission was 5 nm [21].

### 2.7. Antioxidant Activity

#### 2.7.1. DPPH• Scavenging Capacity

The antioxidant activities of Res-loaded HGNPs were evaluated by DPPH• scavenging assay based on the way of Huang et al. (2017) [22]. In short, 2 mL of DPPH• ethanol solution (0.1 mM) was mingled with 2 mL of Res-loaded HGNPs’ dispersion at different concentrations of Res (10~50 μg/mL). After 30-min incubation in the dark, the absorbance of the reaction solution at 517 nm was determined. A total of two controls were measured for comparison, including the ethanol-dissolved Res (free Res) and empty HGNPs. The DPPH• scavenging capacity was calculated by (1):

(1) $$\mathrm{DPPH}•\;\mathrm{scavenging}\;\mathrm{capacity}\;(\%)=\frac{A_{0}–A_{1}}{A_{0}}\times 100\;$$

where *A*_1_ was the absorbance of DPPH radicals after reacting with the sample and *A*_0_ was the absorbance of DPPH radicals without the sample.

#### 2.7.2. ABTS•^+^ Scavenging Capacity

The ABTS•^+^ scavenging capacity was assessed and it referred to the method of Yi et al. (2022) with minor modifications [23]. The ABTS•^+^ aqueous solution (7 mM) and potassium persulfate (4.9 mM) were mixed and reacted for 16 h in the dark to generate the ABTS•^+^ stock solution. The ABTS•^+^ stock was then diluted with phosphate buffer (pH 7.4, 0.01 M) until the absorbance of ABTS•^+^ at 734 nm was 0.70 (±0.02). Then, 4 mL of diluted ABTS•^+^ solution was mingled with 0.1 mL of Res-loaded HGNPs’ dispersion at a Res concentration of 10~50 μg/mL. The reaction was carried out for 6 min in the dark, followed by monitoring absorbance at 734 nm. Free Res and empty HGNPs were regarded as controls. The ABTS•^+^ scavenging capacity was reckoned according to Equation (2):

(2) $$\mathrm{ABTS}{•}^{+}\;\mathrm{scavenging}\;\mathrm{capacity}\;(\%)=\frac{A_{0}–A_{1}}{A_{0}}\times 100\;$$

where *A*_0_ and *A*_1_ were the absorbance of ABTS•^+^ after reacting with and without the sample, respectively.

### 2.8. Encapsulation Efficiency (EE) and Loading Capacity (LC) of Res

The sample solution was mingled with methanol, shaken thoroughly for 30 s and centrifuged at 2000× *g* for 15 min to eliminate unencapsulated Res. The amount of Res in the supernatants (Encapsulated Res) and initial samples (Total Res) was determined by the Chromaster HPLC system (Hitachi, Japan) with LaChrom C18 column (5 μm, 250 × 4.6 mm). The glacial acetic acid/ultrapure water (glacial acetic acid: ultrapure water = 0.2:99.8, *v*/*v*) and acetonitrile (35:65, *v*/*v*) were used as binary mobile phases. Other relevant testing conditions: the temperature was 30 °C, the wavelength was 306 nm and the flow rate of the mobile phase was 1.0 mL/min. Finally, the calibration curve (*y* = 67399*x*, *R*^2^ = 0.9999) was used to calculate the content of Res. The EE and LC were reckoned according to Equations (3) and (4):

(3) $$\mathrm{EE}\;(\%)=\frac{\mathrm{Encapsulated}\;\mathrm{Res}\;(\mathrm{mg})}{\mathrm{Total}\;\mathrm{Res}\;(\mathrm{mg})}\times 100\;$$

(4) $$\mathrm{LC}\;(\%)=\frac{\mathrm{Encapsulated}\;\mathrm{Res}\;(\mathrm{mg})}{\mathrm{Total}\;\mathrm{of}\;\mathrm{Gli}\;\mathrm{and}\;\mathrm{Res}\;(\mathrm{mg})}\times 100\;$$

### 2.9. Morphology by Transmission Electron Microscope (TEM)

The sample was dripped on a copper grid for 3 min, followed by dyeing with uranyl acetate dye for 2 min. After drying, the morphology of gliadin nanoparticles was observed using TEM (JEM-1200, JEOL Ltd., Tokyo, Japan). 

### 2.10. Photostability 

The photostability of Res was assessed based on our previous method [4]. In brief, 2 mL of fresh samples were placed into 2 mL of transparent Eppendorf tubes for uninterrupted ultraviolet radiation at room temperature. The remaining content of Res at a specified time was measured by the HPLC as delineated in Section 2.8. Free Res was used as the control. The retention rate of Res was calculated as follows:

(5) $$\mathrm{Retention}\;\mathrm{rate}\;\mathrm{of}\;\mathrm{Res}\;(\%)=\frac{\mathrm{Remaining}\;\mathrm{content}\;\mathrm{of}\;\mathrm{Res}}{\mathrm{Initial}\;\mathrm{content}\;\mathrm{of}\;\mathrm{Res}}\times 100\;$$

### 2.11. TURBISCAN Stability

The TURBISCAN stability of samples was measured by the TurbiscanLAB instrument (Formulaction, Toulous, France). In brief, 10 mL of sample was taken into a transparent glass vial and scanned every 30 min using the TurbiscanLAB instrument at 25 °C for a specific length of time. The Turbiscan stability index (TSI) value was negatively correlated with the stability of the sample. The TSI value was calculated using Equation (6):

(6) $$\mathrm{TSI}=\sqrt{\frac{\sum_{i\;=1}^{n}{\left(x_{i}–x_{BS}\right)}^{2}}{n\;–1}}$$

where *n* represented the number of scans, *x_i_* was the average amount of backscatter per minute and *x_BS_* was the mean *x_i_*.

### 2.12. In Vitro Release of Resveratrol

The kinetic release profile of Res in the simulated gastrointestinal tract was conducted with reference to the way of Pu et al. (2020) [18]. The simulated gastric fluid (SGF, with 2 mg/mL NaCl and 1.6 mg/mL pepsin, pH 1.2), as well as the simulated intestinal fluid (SIF, with 3.2 mg/mL pancreatin, 10 mg/mL of bile salt, 6 mg/mL of Tris and 1.1 mg/mL of anhydrous CaCl_2_, pH 7.5) were referred to the simulated gastrointestinal tract model that we reported previously [24]. After 10 mL of how the freshly prepared sample was mingled with an equal volume of SGF, the pH of the mixture was regulated to 1.2 and shaken at 150 rpm in 37 °C for 2 h. Subsequently, the resulting gastric digesta was adjusted to pH 7.5 to inactivate pepsin and then mingled with the equivoluminal SIF. The resulting dispersion was shaken at 150 rpm at 37 °C for 4 h to simulate intestinal digestion. At pre-determined times, 1 mL of sample digesta was taken out and replaced with equivoluminal fresh digestive fluid. After centrifugation (10,000 rpm, 5 min), the syringe filter of 0.22 μm was used to percolate the supernatant. The content of Res was measured by the HPLC. The cumulative release rate of Res was reckoned according to Equation (7):

(7) $$\mathrm{Cumulative}\;\mathrm{release}\;\mathrm{rate}\;\mathrm{of}\;\mathrm{Res}\;(\%)=\frac{R_{1}}{R_{0}}\times 100$$

where *R*_0_ was the initial amount of Res in nanoparticles and *R*_1_ represents the cumulative amount of Res released from nanoparticles at the specified time. 

### 2.13. Data Analysis

All data were collected from at least triplicate determinations. Values were indicated as means ± standard deviations. Analysis of the variance was conducted by IBM SPSS 25 software, followed by Duncan’s test at a confidence level of 95%. Origin 2019 was used to generate graphs.

## 3. Results and Discussion

### 3.1. Characterization of Hollow Gliadin Nanoparticles (HGNPs) and Resveratrol-Loaded Hollow Gliadin Nanoparticles (Res-Loaded HGNPs)

Particle size is a vital factor influencing the delivery performance of a nanoparticle, which determines the penetration ability of the nanoparticle in tissues and the absorption rate of the nanoparticle in cells. Considering that the added amount of gliadin (Gli) might affect the particle sizes of HGNPs, the effect of the Gli/Na_2_CO_3_ mass ratio on the particle size of HGNPs was explored. As shown in Figure S1, HGNPs possessed the smallest particle size (121.56 nm) when the Gli/Na_2_CO_3_ mass ratio was 5:1, suggesting that this mass ratio might be the most appropriate. With the improvement of the Gli/Na_2_CO_3_ mass ratio, the particle sizes of HGNPs also gradually increased. A plausible explanation for this result was that more Gli molecules precipitated on the surface of Na_2_CO_3_ templates, leading to a thicker Gli shell. However, as the Gli/Na_2_CO_3_ mass ratio decreased, the particle sizes of HGNPs also increased, which might be due to the fact that there was not enough Gli to coat the Na_2_CO_3_ particles, resulting in the spontaneous aggregation and growth of the Na_2_CO_3_ particles. Therefore, the Gli/Na_2_CO_3_ mass ratio of 5:1 was the optimum ratio for the preparation of HGNPs.

**Figure 1:**
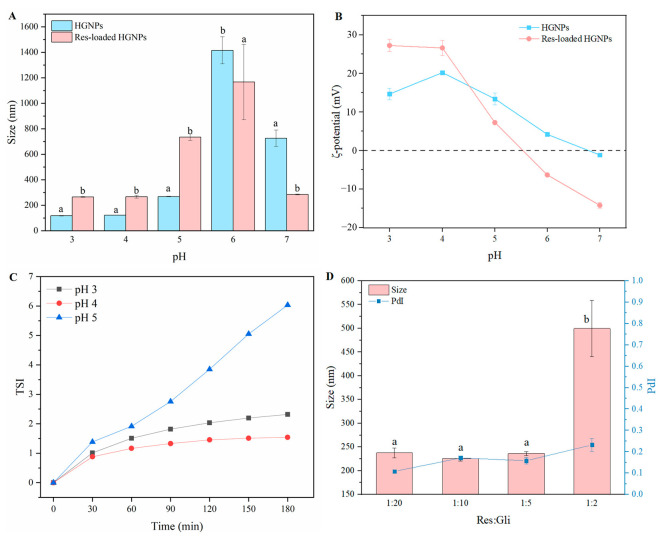
Effect of the pH on particle sizes (**A**) and ζ-potential (**B**) of hollow gliadin nanoparticles (HGNPs) and resveratrol-loaded hollow gliadin nanoparticles (Res-loaded HGNPs). At the same pH, different letters indicate significant differences (*p* < 0.05); (**C**) The TSI of Res-loaded HGNPs at a different pH; (**D**) Particle sizes of Res-loaded HGNPs at different ratios of gliadin to resveratrol (*w*/*w*). Different letters indicate significant differences (*p* < 0.05).

Under the condition of the Gli/Na_2_CO_3_ mass ratio of 5:1, the particle sizes (Figure 1A) and ζ-potential (Figure 1B) of HGNPs and Res-loaded HGNPs at a different pH were further investigated. The results showed that the HGNPs had small particle sizes at pH 3.0, 4.0 and 5.0. When the pH reached 6.0 and 7.0, the particle size of HGNPs increased abruptly, which was due to the fact that the isoelectric point (pI) of the Gli was 6.5 [25]. The results of ζ-potential corresponded to those of particle sizes. The ζ-potential of HGNPs was 4.17 mV at pH 6.0, while the ζ-potential of HGNPs was −1.16 mV at pH 7.0, manifesting the pI of gliadin that was between 6.0 and 7.0. The absolute value of ζ-potential was less than 10 mV, which was not sufficient to prevent the aggregation between HGNPs, so the particle size of HGNPs increased at pH 6.0 and pH 7.0. The particle sizes of Res-loaded HGNPs showed an increasing trend compared to HGNPs, manifesting that Res was successfully loaded into HGNPs, which might be the result of hydrophobic interaction between resveratrol (Res) and Gli. However, the measured particle sizes of Res-loaded HGNPs were smaller than that of HGNPs at pH 7.0. It could be seen from the appearance of Figure S2 that when the pH was 7.0, the Res-loaded HGNPs dispersion became clarified, indicating that Res-loaded HGNPs have been aggregated or even precipitated under this condition, so the particle size measured by the dynamic light scattering technique could not reflect the true particle sizes of nanoparticles. Compared with HGNPs, the ζ-potential of Res-loaded HGNPs increased in an acidic environment (pH 3.0 and pH 4.0), which could be attributed to the Gli molecule exposing more positively charged groups due to the combination of Res and Gli. The large electrostatic repulsion could prevent the aggregation of Res-loaded HGNPs, so Res-loaded HGNPs could exist stably at pH 3.0 and 4.0. In addition, in a neutral environment, the ζ-potential of Res-loaded HGNPs increased, which might be due to the ionization of the phenol hydroxyl group on Res.

To further investigate the optimal pH, the Turbiscan stability index (TSI) values were tested to assess the TURBISCAN stability of Res-loaded HGNPs at a pH of 3.0, 4.0 and 5.0. The TSI value is negatively correlated with the stability of the sample, which means that the lower TSI value signifies the greater stability of the sample. As seen in Figure 1C, the TSI values of Res-loaded HGNPs at pH 5.0 were higher than those at pH 3.0 and pH 4.0, which indicated Res-loaded HGNPs at pH 5.0 were less stable. A reasonable explanation for this result was that pH 5.0 was closer to the pI of Gli. At pH 5.0, the particle size of Res-loaded HGNPs was larger and the absolute value of ζ-potential was smaller. Besides, the TSI values of Res-loaded HGNPs at pH 3.0 were higher than those at pH 4.0, indicating that Res-loaded HGNPs were more unstable at pH 3.0, which might be due to the fact that the peptide chains of Gli were partially expanded at pH 3.0, exposing more hydrophobic regions [26]. The hydrophobic interaction between Res-loaded HGNPs was stronger, resulting in hydrophobic aggregation among Res-loaded HGNPs. Therefore, in subsequent studies, pH 4.0 was chosen as the optimal pH for preparing Res-loaded HGNPs. Finally, the effect of the Res/Gli mass ratio on the particle size of Res-loaded HGNPs was explored. As displayed in Figure 1D, the particle size of Res-loaded HGNPs was 236.20 nm as the Res/Gli mass ratio was 1:5. When the Res/Gli mass ratio was further reduced (Res/Gli = 1:10 or 1:20), the particle size of Res-loaded HGNPs did not change significantly and all were less than 250 nm. In addition, the polydispersity index (PdI) was less than 0.2 at a Res/Gli mass ratio in the range of 1:20 to 1:5, suggesting that Res-loaded HGNPs were uniform and stable. However, when the Res/Gli mass ratio increased to 1:2, the particle sizes of HGNPs were remarkably increased (*p* < 0.05) to around 500 nm. This might be due to the fact that the content of Res exceeded the maximum encapsulation capacity of HGNPs and the superfluous Res might facilitate the aggregation of HGNPs. After further increasing the ratio of Res/Gli (above 1:5), it was found that the prepared nanoparticle possessed a too large particle size, poor stability and significantly reduced encapsulation efficiency. Many similar studies have also reported that excess bioactive substances could lead to increased particle size and reduced stability [27,28].

### 3.2. Encapsulation Efficiency (EE) and Loading Capacity (LC)

Table 1 showed the EE and LC of Res at different Res/Gli mass ratios. The results revealed that the EE of Res decreased with the increase of the Res/Gli mass ratio, which was due to the fact that as Res/Gli mass ratio increases, superabundant Res precipitates on the surface of the nanoparticles or is suspended in the solution instead of being loaded inside the nanoparticles. A similar tendency in EE has been reported with kafirin-based hollow nanoparticles prepared through the deposition of dextran sulfate/chitosan, which was used to deliver curcumin [20]. As the Res/Gli mass ratio was enhanced from 1:20 to 1:2, the EE of Res in HGNPs decreased from 95.38 to 49.69%, which was higher than that in SGNPs where EE reduced from 94.40 to 14.73%. As for the LC of Res, it increased with the increase of the Res/Gli mass ratios, which could be attributed to the improvement in the proportion of Res. Compared with Res-loaded SGNPs, Res-loaded HGNPs revealed a higher LC of Res, which might be due to the fact that hollow structures of HGNPs could encapsulate and load more of the Res. From the digital images (Figure S3) of Res-loaded HGNPs and Res-loaded SGNPs at different Res/Gli mass ratios, it could be seen that the color of nanoparticle dispersions became deeper with the increase in the Res/Gli mass ratio. The remarkable decrease in the EE of Res at a Res/Gli mass ratio of 1:2 might manifest that the content of Res was oversaturated at this mass ratio, resulting in the precipitation of nanoparticles, which was consistent with the phenomenon shown in the photograph (Figure S3).

### 3.3. Morphological Observation

**Figure 2:**
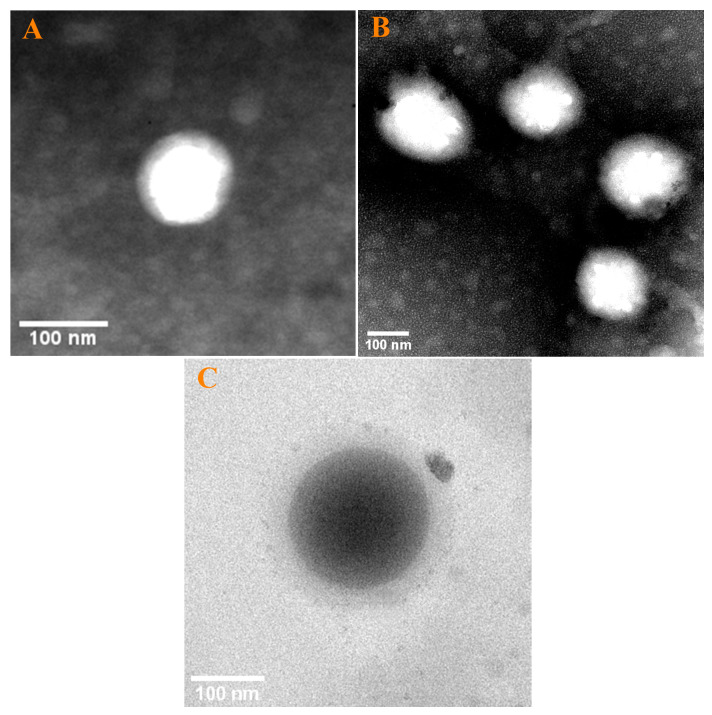
TEM images of HGNPs (**A**), Res-loaded HGNPs (**B**) and SGNPs (**C**).

The microstructure of the nanoparticle was observed by TEM, as shown in Figure 2. From the perspective of the particle’s structure, both HGNPs (Figure 2A) and Res-loaded HGNPs (Figure 2B) displayed distinct core-shell structures, where the light-colored region represented the hollow core and the dark rim represented the shell, indicating that the encapsulate of Res did not change the core-shell structure of HGNPs. Furthermore, the particle size of Res-loaded HGNPs was larger than that of HGNPs, proving that Res was successfully loaded into the HGNPs, which was consistent with the results determined by the DLS technique. Figure 2C showed the TEM image of SGNPs, which indicated that SGNPs possess an obviously solid structure. The black shadow in SGNPs is attributable to the fact that the solid structure of SGNPs can block more electrons. In addition, it was found that the particle sizes observed by TEM were generally smaller than those by DLS measurements, which was owing to the shrinkage of nanoparticles during the drying process prior to TEM observation.

### 3.4. FTIR Analysis

**Figure 3:**
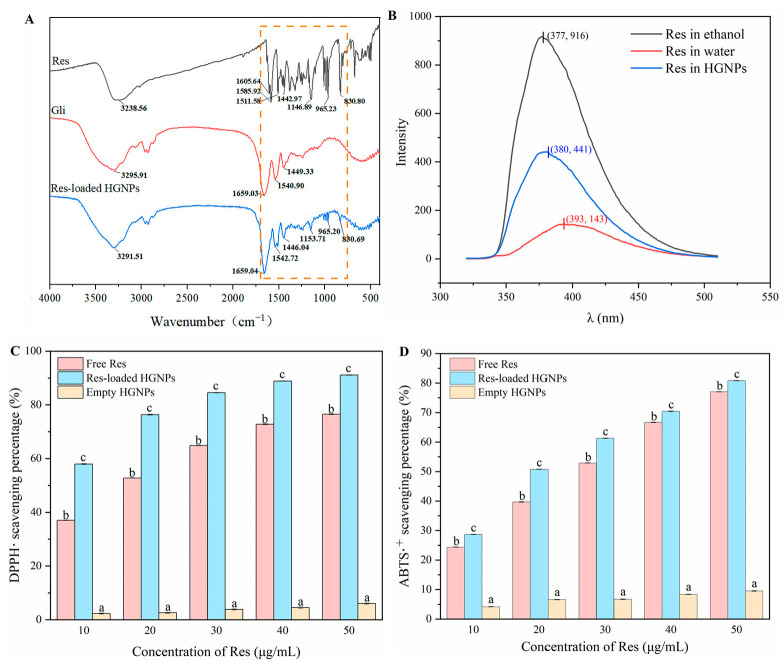
FTIR spectra (**A**) of resveratrol (Res), gliadin, Res-loaded hollow gliadin nanoparticles (Res-loaded HGNPs); (**B**) Fluorescence emission spectra of Res in water, ethanol and HGNPs; DPPH• scavenging capacity (**C**) and ABTS•^+^ scavenging capacity (**D**) of free Res, Res-loaded HGNPs and empty HGNPs. At the same concentration of Res, different letters indicate significant differences (*p* < 0.05).

FTIR spectrum can reveal information about the functional group as well as the chemical bond. The FTIR spectra of different samples were depicted in Figure 3A. The broad peak of 3200 cm^−1^~3550 cm^−1^ corresponded to the stretching vibrations of −OH. The FTIR spectra of Res showed many characteristic peaks, where the peaks at 1605.64 cm^−1^, 1511.58 cm^−1^ and 1442.97 cm^−1^ were concerned with the stretching vibrations of the aromatic −C=C−. The peaks that appeared at 1585.92 cm^−1^, 1146.89 cm^−1^ and 965.23 cm^−1^ were associated with −C−O− stretching, olefinic −C−C− stretching as well as trans olefinic −C=C− bending vibrations, respectively. For Gli, typical amide I and amide II absorption peaks were located at 1659.03 cm^−1^ and 1540.90 cm^−1^, respectively. Absorption peaks at 1449.33 cm^−1^ were related to −CH_2_ bending vibrations. For Res-loaded HGNPs, the presence of characteristic peaks of Res at 1153.71 cm^−1^, 965.20 cm^−1^ and 830.69 cm^−1^ proved the existence of Res. However, the absorption intensity of the characteristic peaks was significantly reduced and some characteristic peaks of Res vanished (circled by dotted lines in Figure 3A), indicating that the Res was wrapped in the hydrophobic core of the nanoparticle, which limited the stretching and bending of various bonds of the Res. Furthermore, the peaks of −OH were shifted to 3291.51 cm^−1^, which might be owing to the formulation of hydrogen bonds between amide groups of glutamine in Gli and phenolic groups in the Res [8]. Due to the hydrophobic characteristics of the Res and Gli, the hydrophobic interaction was the other driving force forming Res-loaded HGNPs.

### 3.5. Fluorescence Spectroscopy Analysis

The fluorescence intensity of polyphenols was related to their surrounding microenvironment. As seen in Figure 3B, the fluorescence intensity of Res in ethanol was 6.4 times higher than that in water. Furthermore, the spectrum of Res had an emission maximum (*λ*_max_) at 393 nm and 377 nm in water as well as ethanol, respectively. The increase in the fluorescence intensity and the blue shift of *λ*_max_ could be attributed to the decrease in the polarity of the microenvironment surrounding Res [21]. The fluorescence of Res wrapped in HGNPs exhibited the strongest fluorescence intensity at 380 nm, which was 3.0 times higher than that in water. The reason for this phenomenon was that the non-radiative attenuation of energy decreased and the binding of Res to the hydrophobic points of Gli limited its mobility, which also indicated that a fraction of Res was embedded in the hydrophobic shell of Gli [29]. In addition, the microenvironment of Res encapsulated in HGNPs was less hydrophobic than that in ethanol, as such, the fluorescence intensity of Res in HGNPs was lower than that in ethanol.

### 3.6. Antioxidant Activity

The DPPH• and ABTS•^+^ scavenging capacities of Res-loaded HGNPs were examined to evaluate their antioxidant activities. The antioxidant activities of free Res in ethanol solution and empty HGNPs were also compared with those of Res-loaded HGNPs under the same conditions. The DPPH• and ABTS•^+^ scavenging activity of Res at different concentrations were investigated (Figure 3C,D). As the concentration of Res increased from 10 μg/mL to 50 μg/mL, the DPPH• scavenging rate of free Res increased from 37.02% to 76.53% and the ABTS•^+^ scavenging rate increased from 24.38% to 77.05%. For Res-loaded HGNPs, the DPPH• scavenging rate increased from 57.97% to 91.11% and the ABTS•^+^ scavenging rate increased from 28.67& to 80.81%. Meanwhile, the DPPH• scavenging rate of empty HGNPs increased from 2.32% to 6.01% and the ABTS•^+^ scavenging rate increased from 4.13% to 9.52%. The radical scavenging activities of both free Res and Res-loaded HGNPs were concentration-dependent, with a gradual increase in scavenging activity as the Res concentration increased. In comparison with free Res, Res-loaded HGNPs showed a significantly higher DPPH• and ABTS•^+^ scavenging ability at all measured concentrations. The possible explanations for the results were that the surface area of Res-loaded HGNPs was larger and that the dispersibility of encapsulated Res was apparently improved, which jointly promoted the kinetics of the reaction with free radicals [30]. Furthermore, the Res-loaded HGNPs showed a higher antioxidant activity than the free Res at the same concentration, which was mainly due to the fact that empty HGNPs also exhibited a certain antioxidant capacity. The phenolic hydroxyl groups on Res could provide the hydrogen atoms to scavenge DPPH• and ABTS•^+^. According to the previous report, certain specific amino acids’ residues (including tyrosine, phenylalanine and tryptophan) in protein were able to reactivate the antioxidant activity of Res by providing hydrogen atoms on phenolic rings [23]. The results of all samples in ABTS•^+^ scavenging capacity performed an analogical tendency to the DPPH• scavenging capacity. In short, these results suggested that Res-loaded HGNPs were impactful delivery systems for improving the DPPH• and ABTS•^+^ scavenging capacity of the Res. Similarly, other research had reported that nano-encapsulation strengthened the radical scavenging capacity of bioactive ingredients [22,31].

### 3.7. Comparison of Properties between HGNPs and SGNPs

#### 3.7.1. Structural Difference

**Figure 4:**
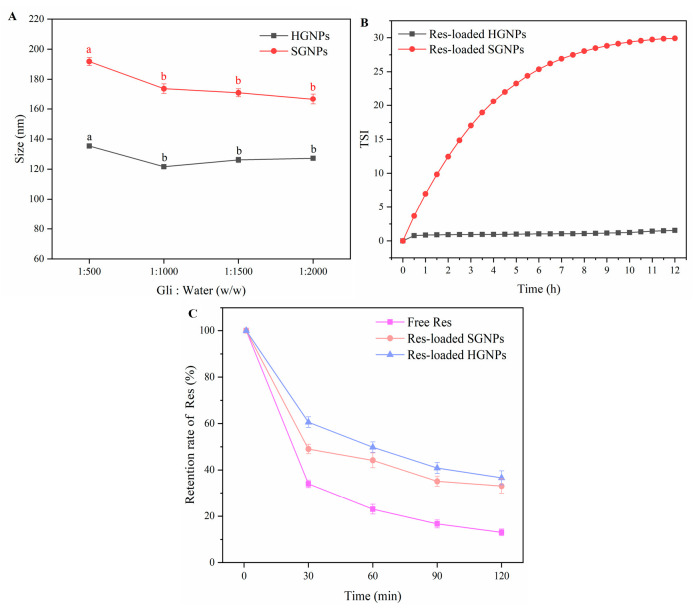
(**A**) The particle size of HGNPs and SGNPs at different weight ratios of gliadin to water The same sample with different letters indicate significant differences (*p* < 0.05).; (**B**) the Turbiscan Stability Index (TSI) of resveratrol-loaded hollow gliadin nanoparticles (Res-loaded HGNPs) and solid gliadin nanoparticles (Res-loaded SGNPs); (**C**) the retention rate (%) of free Res, Res-loaded HGNPs and Res-loaded SGNPs after ultraviolet light irradiation.

The TEM image visualized the structural differences between HGNPs and SGNPs. Unlike the hollow structure of HGNPs (Figure 2A), SGNPs (Figure 2C) appeared to have a distinctly solid structure. As seen in the image, the particle sizes of HGNPs were smaller than those of SGNPs, which was mainly due to the difference in the preparation methods of the two types of nanoparticles. In contrast to the spontaneous nucleation of Gli in water to form SGNPs, the abundance of pre-formed Na_2_CO_3_ nanocrystals served as heterogeneous nucleation sites during the preparation of HGNPs, preventing the increase in the particle size and resulting in the relatively small particle size of HGNPs. The previously reported hollow zein and hollow kafirin nanoparticles also manifested a significant reduction in size compared to their solid nanoparticles [20,32]. For further verification, the particle sizes of HGNPs and SGNPs were also measured by the DLS technique (Figure 4A), which was in conformity with the results observed by TEM. The results revealed the average sizes of HGNPs were smaller than that of SGNPs at all ratios of Gli to water (*w*/*w*), which could be attributed to the presence of Na_2_CO_3_ nanocrystals. Furthermore, the average sizes of HGNPs decreased significantly from 135.40 nm to 121.57 nm whereas that of SGNPs decreased from 191.80 mn to 173.73 nm when Gli/water weight ratios were decreased from 1:500 to 1:1000. Due to the increase of water, the concentration of Gli in the solution decreased, thus, decreasing the possible agglomeration and leading to a reduction in the particle size of nanoparticle [32]. 

#### 3.7.2. Stability Difference

To compare the TURBISCAN stability of Res-loaded HGNPs and Res-loaded SGNPs, their TSI values were determined. The lower TSI values indicated the better stability of the samples. The TSI values of Res-loaded HGNPs were remarkably lower than those of the Res-loaded SGNPs (Figure 4B), implying that the Res-loaded HGNPs were more stable. This might be due to the fact that the hollow nanoparticles possessed smaller particle sizes than solid nanoparticles. From a thermodynamics perspective, Gli polymers readily aggregated into large-sized particles to decrease the surface free energy during the phase transition from dissolution to a solid state [33]. Therefore, the solution containing nanoparticles with larger particle sizes generally exhibited greater instability during storage.

#### 3.7.3. Resistance Difference to Ultraviolet Light

As we all know, Res possesses strong antioxidant activity, however, its application is limited by its sensitivity to ultraviolet (UV) light. Therefore, it is vital to maintain its photostability during storage. The retention rates of Res were determined (Figure 4C). The results showed that after 30 min of UV irradiation, the retention rates of free Res, Res entrapped in SGNPs and Res entrapped in HGNPs decreased rapidly to 34.03%, 49.01% and 60.61%, respectively. After 2 h, the retention rate of free Res decreased to 13.01%; the retention rate of Res entrapped in SGNPs reduced to 33.05%; and the retention rate of Res entrapped in HGNPs reduced to 36.60%. It was found that the photodegradation of Res was a rapid process. The retention rate of Res dropped rapidly within 30 min, while during the subsequent 30~120 min, the retention rate of Res decreased slowly with the extension of light time. The Res entrapped in SGNPs and HGNPs performed better with stability against UV irradiation in comparison with free Res. The result might be attributed to that the Gli nanoparticles provided a physical barrier and shielding effect for Res against UV irradiation. Certain specific amino acids and double bonds contained in the Gli molecules had the capability to absorb UV irradiation [34]. In addition, the retention rate of Res entrapped in HGNPs was higher than that in SGNPs, suggesting that HGNPs performed better protection for Res than SGNPs. The main reason for this result was the different microstructure of nanoparticles. Specifically, in the case of HGNPs, Res is mainly encapsulated in hollow cavities of nanoparticles and protected by the Gli shell, effectively blocking UV irradiation. For SGNPs, mostly Res is adsorbed on the surface of nanoparticles and is more susceptible to photodegradation when directly exposed to UV irradiation.

### 3.8. In Vitro Release Study

**Figure 5:**
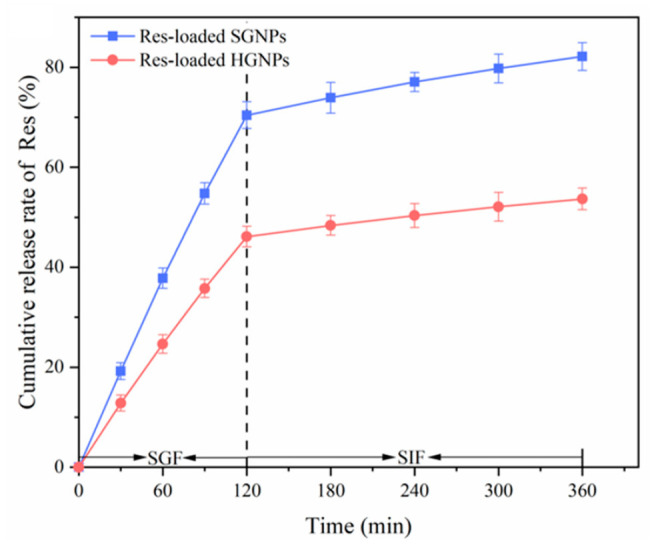
Resveratrol release profile of resveratrol-loaded hollow gliadin nanoparticles (Res-loaded HGNPs) and resveratrol-loaded solid gliadin nanoparticles (Res-loaded SGNPs).

The release profiles of Res loaded in HGNPs and SGNPs in the simulated gastrointestinal tract were depicted in Figure 5. For both HGNPs and SGNPs, Res exhibited a rapid release in the simulated gastric fluid (SGF), which could be attributed to the swelling and partial breakdown of Gli nanoparticles in an acidic environment. After digestion in SGF for 2 h, 46.1% of Res loaded in HGNPs were released into the medium, compared to 70.4% of Res released from SGNPs. The main reason for this discrepancy was that Res adsorbed on the surface of SGNPs was rapidly shed and released into the SGF, whereas Res trapped in the hollow core of HGNPs was released when HGNPs were swelled and decomposed in an acidic environment. The result demonstrated that the HGNPs manifested a stronger protective effect compared to SGNPs in inhibiting the premature release of Res in the simulated gastric stage. In the simulated intestinal fluid (SIF), the release rate of Res was remarkably reduced due to the mild environment of the intestine. At the end of SIF, the cumulative release rate of Res from SGNPs and HGNPs reached 82.1% and 53.6%, respectively. The release rate of Res loaded in HGNPs was slower than that loaded in SGNPs, which indicated that HGNPs could postpone the release of Res and exhibited a better sustained-release effect during the simulated intestinal period. According to the report, Res was primarily metabolized in the liver and intestine via enterohepatic circulation [32]. Therefore, reducing the release of Res in the stomach and prolonging the release of Res in the intestine were conducive to improving the bioavailability of Res. A similar phenomenon was found that curcumin-loaded kafirin hollow nanoparticles prepared through the deposition of dextran sulfate/chitosan manifested a sustained-release of curcumin during the simulated gastrointestinal stage in comparison to solid nanoparticles [20].

**Figure 6:**
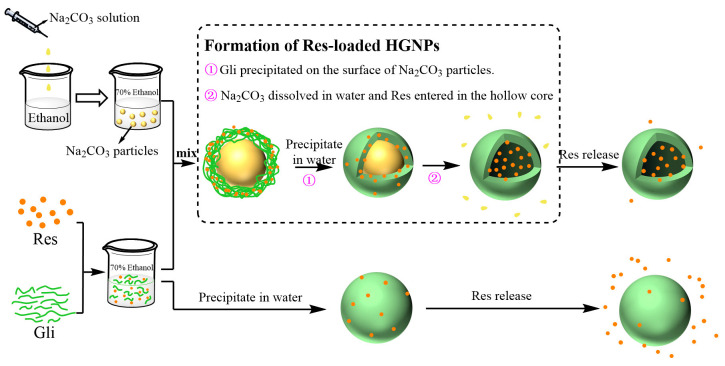
Schematic diagram for the formation and release mechanisms of Res-loaded HGNPs and Res-loaded SGNPs.

This difference in results could be explained by the structural difference between the two types of nanoparticles. The schematic illustration for the formation and release mechanism of Res-loaded HGNPs and Res-loaded SGNPs was shown in Figure 6. The formation progress of Res-loaded HGNPs can be divided into two steps: (i) Gli is deposited on the surface of Na_2_CO_3_ particles due to anti-solvent precipitation, forming the Gli shell; (ii) when water diffuses into the core, the original Na_2_CO_3_ core dissolves due to its good water solubility, while Res in the Gli shell enters the hollow cavity due to the hydrophobic effect, thus, forming Res-loaded HGNPs spontaneously. For HGNPs, Res was entrapped inside the hollow core of nanoparticles; while for SGNPs, Res was partially encapsulated inside the nanoparticles and partially adsorbed on the surface of nanoparticles. Therefore, Res loaded in SGNPs was more readily released during simulated digestion. This also suggested that the hollow structure of HGNPs was conducive to the sustained release of Res, thus, allowing more Res to be absorbed by the human body during the intestine and subsequent digestive stages.

## 4. Conclusions

In conclusion, the hollow gliadin nanoparticles (HGNPs) were successfully fabricated using sodium carbonate as the sacrificial template. When HGNPs were used as delivery carriers of resveratrol (Res), it was found that the optimum preparation conditions for Res-loaded hollow gliadin nanoparticles were determined as a Res to gliadin mass ratio of 1:5 at pH 4.0. Hydrophobic interactions and hydrogen bonds were the prime driving forces to form Res-loaded HGNPs. The antioxidant experiments manifested the antioxidant capacity of Res-loaded HGNPs that were significantly higher than that of free Res. The comparison of two types of gliadin nanoparticles (hollow and solid) showed that HGNPs possessed smaller particle sizes, better stability, higher encapsulation efficiency (93.42%) and loading capacity (15.97%). It was worth mentioning that the hollow structure of HGNPs was more conducive to the sustained release of Res in the gastrointestinal tract. The HGNPs developed in this study may be a promising and ideal delivery carrier of bioactives applied in functional beverages and foods. In the future, the effect of HGNPs on celiac disease can be investigated and further in vivo animal studies are needed to accurately evaluate the stability, safety and functionality of the hollow protein-based nanoparticle delivery system.

## Figures and Tables

**Table 1:** The EE and LC of Res loaded in hollow gliadin nanoparticles (HGNPs) and solid nanoparticles (SGNPs) at different Res/Gli mass ratios (1:2, 1:5, 1:10 and 1:20).

Res: Gli (*w*/*w*)	EE (%)		LC (%)	
	HGNPs	SGNPs	HGNPs	SGNPs
1:2	49.69 ± 0.80 ^a^	14.73 ± 0.24 ^a^	16.56 ± 0.63 ^d^	4.91 ± 0.08 ^b^
1:5	93.42 ± 0.23 ^b^	82.25 ± 0.07 ^b^	15.97 ± 0.27 ^c^	13.71 ± 0.11 ^d^
1:10	95.14 ± 0.25 ^c^	94.11 ± 0.40 ^c^	8.65 ± 0.34 ^b^	8.56 ± 0.04 ^c^
1:20	95.38 ± 0.17 ^c^	94.40 ± 0.52 ^c^	4.54 ± 0.24 ^a^	4.50 ± 0.02 ^a^

Note: Different letters in each column show significant differences among mean values (*p* < 0.05).

## Data Availability

The data used to support the findings of this study can be made available by the corresponding author upon request.

## Supplementary Materials

The supporting information can be downloaded at: https://www.mdpi.com/article/10.3390/foods12132436/s1, Figure S1: effect of mass ratios of gliadin (Gli) to Na_2_CO_3_ on particle sizes of hollow gliadin nanoparticles; Figure S2: the digital images of resveratrol-loaded hollow gliadin nanoparticles at different pH values; Figure S3: the digital images of resveratrol-loaded hollow gliadin nanoparticles (Res-loaded HGNPs) and resveratrol-loaded solid gliadin nanoparticles (Res-loaded SGNPs) at different Res/Gli mass ratios.
