# Supplementary material for: Development, Characterization and Resveratrol Delivery of Hollow Gliadin Nanoparticles: Advantages over Solid Gliadin Nanoparticles
Source: Foods. 2023 Jun 21;12(13):2436. doi: 10.3390/foods12132436 (PMC10340566; doi:10.3390/foods12132436)
Supplement: Supplementary file 1 [file foods-12-02436-s001.zip › foods-2414123-supplementary.pdf]

Supplementary Information For

Development, Characterization and Resveratrol Delivery of Hollow Gliadin Nanoparticles: Advantages over Solid Gliadin Nanoparticles

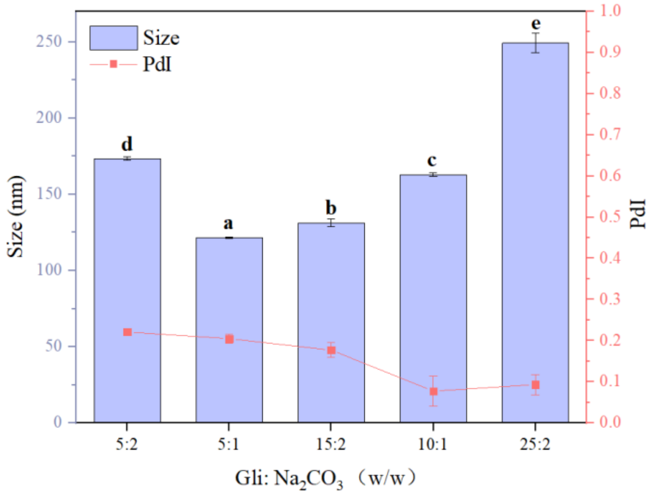

Figure S1. Effect of mass ratios of gliadin (Gli) to Na_2CO_3 on particle sizes of hollow gliadin nanoparticles.

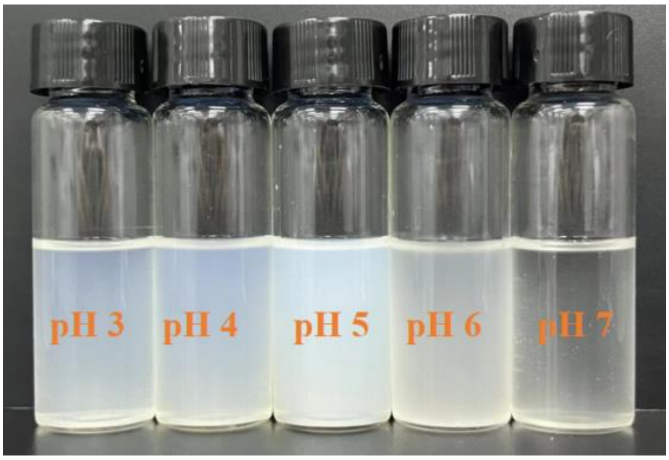

Figure S2. The digital images of resveratrol-loaded hollow gliadin nanoparticles at different pH values.

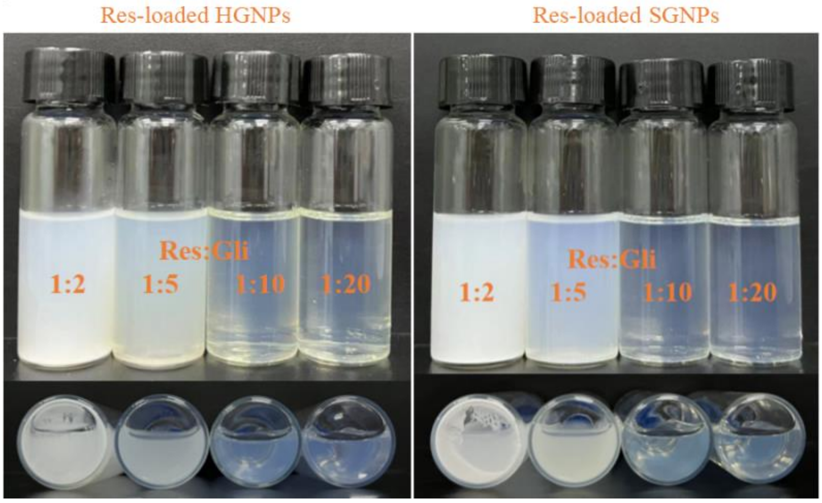

Figure S3. The digital images of resveratrol-loaded hollow gliadin nanoparticles (Res-loaded HGNPs) and resveratrol-loaded solid gliadin nanoparticles (Res-loaded SGNPs) at different mass ratios of resveratrol (Res) to gliadin (Gli).
